# Multistate Outbreak of *Salmonella enterica* Serovar Heidelberg with Unidentified Source, Australia, 2018–2019

**DOI:** 10.3201/eid2801.211462

**Published:** 2022-01

**Authors:** Elenor J. Kerr, Russell Stafford, Irani U. Rathnayake, Rikki M.A. Graham, Emily Fearnley, Joy Gregory, Keira Glasgow, Rose Wright, Vitali Sintchenko, Qinning Wang, Peter Howard, Lex E.X. Leong, Mary Valcanis, William Pitchers, Stephen B. Lambert, Amy V. Jennison

**Affiliations:** Queensland Health, Brisbane, Queensland, Australia (E.J. Kerr, R. Stafford, I.U. Rathnayake, R.M.A. Graham, S.B. Lambert, A.V. Jennison);; Australian National University, Canberra, Australian Capital Territory, Australia (E.J. Kerr, S.B. Lambert);; South Australia Health, Adelaide, South Australia, Australia (E. Fearnley);; Department of Health and Human Services, Melbourne, Victoria, Australia (J. Gregory);; Health Protection New South Wales, St. Leonard’s, New South Wales, Australia (K. Glasgow);; Department of Health, Canberra (R. Wright);; Institute of Clinical Pathology and Medical Research, Westmead, New South Wales, Australia (V. Sintchenko, Q. Wang, P. Howard);; South Australia Pathology, Adelaide (L.E.X. Leong);; University of Melbourne, Parkville, Victoria, Australia (M. Valcanis, W. Pitchers)

**Keywords:** salmonella, Australia, epidemiology, disease outbreaks, antimicrobial resistance, bacteria, food safety, *Salmonella enterica*, food poisoning, enteric infections

## Abstract

We report a multistate *Salmonella* *enterica* serovar Heidelberg outbreak in Australia during 2018–2019. Laboratory investigation of cases reported across 5 jurisdictions over a 7-month period could not identify a source of infection but detected indicators of severity and invasiveness. The hospitalization rate of 36% suggested a moderately severe clinical picture.

*Salmonella enterica* serovar Heidelberg is a frequently identified serotype among infections in humans in North America, East Africa, and Asia but is uncommon in Australia. An average of 37 cases of *Salmonella* Heidelberg were notified in Australia annually in 2009–2017, predominantly overseas acquired (*1*). Six outbreaks have been reported nationally since 1995; 1 outbreak in 1996 had >500 cases, but most have <7 cases (R. Bell, pers. comm. [email], 2020 Jun 16). We report a national outbreak of *Salmonella* Heidelberg infection across 5 jurisdictions over 7 months.

## The Study

In December 2018, OzFoodNet, Australia’s government-based network for enhanced foodborne disease surveillance, noted that *Salmonella* Heidelberg cases diagnosed in November (15 cases) were above the national historical 5-year mean (2.4 cases). New South Wales (NSW) and Victoria initiated separate investigations during December 2018–February 2019; neither developed a hypothesis regarding potential sources of infection. In February–March 2019, whole-genome sequencing (WGS) analysis of available isolates identified 36 highly related cases, 12 each from Queensland, NSW, and Victoria. Concurrent *Salmonella* Heidelberg infections with WGS pending were subsequently identified in other states: South Australia (SA) (N = 4) and Western Australia (WA) (N = 3). Queensland cases were not initially investigated because *Salmonella* Heidelberg is more common in this state; Queensland contributed 43% of cases in Australia during 2009–2017 (*1*). Outbreak cases were reported across multiple jurisdictions peaking in early December 2018 and continuing through late March 2019, with an outlying case reported in May 2019 (Figure). After confirmation of phylogenetic relatedness and previous jurisdictional inability to identify a common source, OzFoodNet commenced a multijurisdictional outbreak investigation in March 2019. However, case numbers declined soon after, preventing more rigorous, prospective epidemiologic investigation.

**Figure:**
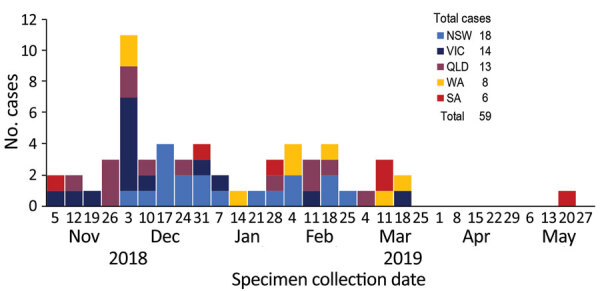
*Salmonella enterica* serovar Heidelberg outbreak cases by week of specimen collection and jurisdiction, Australia, November 2018–May 2019 (n = 59). NSW, New South Wales; QLD, Queensland; SA, South Australia; VIC, Victoria; WA, Western Australia.

We identified 59 outbreak cases in 5 jurisdictions (58 laboratory-confirmed, 1 epidemiologically-linked): NSW (18/59, 31%), Victoria (14/59, 24%), Queensland (13/59, 22%), WA (8/59, 14%), and SA (6/59, 10%) (Table). Case-patients were 2 months–95 (median 43) years of age. None had a history of international travel; 3 case-patients reported interstate travel during their exposure period.

**Table:** National *Salmonella enterica* serovar Heidelberg outbreak cases by demographic and clinical characteristics, Australia, November 1, 2018–July 10, 2019

Feature	No. (%)
Demographic	N = 59
Sex	
M	33 (56)
F	26 (44)
Age group, y	
0–4	10 (17)
5–9	2 (3)
10–19	5 (8)
20–29	2 (3)
30–39	7 (12)
40–49	5 (08)
50–59	12 (20)
60–69	6 (10)
70–79	3 (5)
80–89	5 (8)
≥90	2 (3)
Symptom	
Diarrhea	38/42 (90)
Abdominal cramps	30/37 (81)
Fever	22/38 (58)
Vomiting	11/38 (30)
Bloody diarrhea	7/34 (21)
Hospitalization	
Yes	16/45 (36)
No	29/45 (64)
Emergency department visit only	3/16 (19)

Thirty-nine case-patients completed interviews using telephone-administered hypothesis-generating questionnaires. The remaining 20 patients were not investigated because they refused interviews, they could not be contacted, or their diagnosis dates precluded obtaining a reliable food history. Binomial probability analysis compared case-patient food exposures to background rates estimated from a Victoria population survey, accounting for seasonality, from November 2014–October 2016 (*2*).

Probability calculations highlighted potential foods of interest, including cooked chicken (p<0.001), macadamia nuts (p = 0.001), frozen vegetable products (p = 0.005), and lamb (p = 0.005). Global epidemiology suggests *Salmonella* Heidelberg outbreaks are most likely associated with poultry or eggs (*3**–**5*), yet case reporting of poultry products consumed and place of purchase did not identify a common source. Raw macadamia nuts were considered because of their popularity during the Christmas period, along with previous detections of *Salmonella* Heidelberg in Queensland (*6*). Because epidemiologic evidence was insufficient to develop a strong hypothesis for any single food item, sampling was not considered feasible; decreasing case numbers precluded an analytic study.

We consolidated sequence data, including isolates sequenced in jurisdictional laboratories. We assessed genetic relatedness among isolates of *Salmonella* Heidelberg cases at the Queensland Health Forensic and Scientific Services laboratory by generating core genome multilocus sequence typing (cgMLST) complex types (<7 allele differences) using Ridom SeqSphere+ version 5.1.0 (https://www.ridom.de) based on the EnteroBase *Salmonella* enterica scheme version 2.0 (https://enterobase.warwick.ac.uk). We determined single-nucleotide polymorphism (SNP) differences using Snippy package version 4.3.6 (https://github.com/tseemann/snippy) using *Salmonella* Heidelberg SL476 (GenBank accession no. NC_011083.1) as a reference. We conducted in silico WGS analysis for antimicrobial-resistance genes by the abricate program version 0.8.10 (https://github.com/tseemann/abricate), using the Resfinder database October 18, 2018, version (https://cge.cbs.dtu.dk/services/ResFinder) to further characterize strains. We investigated the presence of the *saf* operon using Ridom SeqSphere+ against the *saf* operon sequence from GenBank (accession no NZ_LS483494). We generated sequences for isolates on the Illumina NextSeq genome sequencing platform (https://www.illumina.com) using the Nextera XT library preparation kit; the WGS read files were deposited in the US National Center for Biotechnology Information (https://www.ncbi.nlm.nih.gov) Sequence Read Archive (BioProject ID PRJNA663036 [NSW] and PRJNA660800 [Queensland, Victoria, SA, WA]). We developed our hypothesis using a range of local and international sequence data sources to assess genetic relatedness to outbreak cases; we used 78 human sequences from 5 jurisdictional laboratories, 8 nonhuman *Salmonella* Heidelberg isolates from a Queensland culture collection from food and animals (macadamia nuts and poultry, caprine, porcine, bovine, equine, and reptile sources), and 86 international sequences downloaded from EnteroBase and the National Center for Biotechnology Information.

The 58 *Salmonella* Heidelberg isolates available from this outbreak were all multilocus sequence type (ST) 15 and belonged to the same cgMLST complex type (2561); isolates differed within the complex type by 0–3 SNPs/cgMLST alleles, whereas the nearest nonoutbreak genomes had >34 SNP and >16 SNP allele differences (Appendix Figure). This complex type was not identified among a range of international sequences or Australia nonhuman or historical human isolates chosen to inform possible outbreak sources; thus, we could not develop our hypothesis using WGS. No compelling microbiological evidence supported hypotheses of a nut, poultry, or other specific source. 

All Australia isolates and 91% of international isolates harbored the antimicrobial-resistance gene *fosA7* for fosfomycin (*7*). Phenotypic analysis of 9 isolates revealed the outbreak strains to be susceptible to antimicrobial drugs including cephalosporins, fluoroquinolones, aminoglycosides, broad-spectrum penicillins, and trimethoprim/sulfamethoxazole.

Of 45 case-patients for whom data were available, 16 (36%) were hospitalized with a median duration of 4 (range 1–18) days, reported by 13 case-patients. Although direct comparison is difficult because of potential confounding by age, the hospitalization rate of 36% was high compared with the rate of 11.6% among 149 US outbreaks of *Salmonella* Heidelberg from 1973–1997 (*3*). Hospitalization rates in that outbreak were also higher than that for *Salmonella enterica* outbreaks in Australia with similar age distributions during 2001–2016 (*8*). 

Although this investigation was unable to capture invasiveness of the outbreak strain, *Salmonella* Heidelberg has frequently been associated with greater risk for invasive disease than have other commonly reported nontyphoidal *Salmonella* serotypes, including Typhimurium and Enteritidis (*9**–**14*). In the United States, *Salmonella* Heidelberg is among the 4 most common serotypes isolated from blood; 12%–13% of *Salmonella* Heidelberg infections resulted in invasive disease in North America, higher than the US *Salmonella* average of 7% (*10**–**12*). A study of invasive nontyphoidal *Salmonella* infection in Australia similarly found that almost 10% of *Salmonella* Heidelberg gastrointestinal infections during 2007–2016 were invasive disease, ≈5× higher than *Salmonella* Typhimurium infections (*15*). Concern has grown regarding the virulence of a US bovine-related *Salmonella* Heidelberg outbreak; recent genomic analyses indicated that most identified *Salmonella* virulence genes are present in most *Salmonella* Heidelberg strains. These studies highlighted potential contributions of *saf* fimbrial genes to increased severity via their role in bacterial aggregation, colonization, and biofilm formation (*9*). The *saf* operon has been reported generally absent from the *Salmonella* Heidelberg serovar but was present in a previous outbreak associated with increased severity (*9*). The *saf* operon was present in all Australia isolates in this study (Appendix Figure).

## Conclusions

We report a national outbreak investigation of a locally uncommon *S. enterica* serovar of unknown origins in Australia. Although *Salmonella* Heidelberg outbreaks are relatively uncommon in Australia, given this outbreak’s comparatively high hospitalization rate and the presence of *saf* fimbrial genes in the implicated strain, future cases warrant prompt investigation to assess severity and invasiveness. A platform for real-time exchange of sequence data in Australia and use of routine WGS for salmonellosis cases, including comparison with local and international strain data, may enable more timely detection of outbreaks.

Appendix Additional information about *Salmonella enterica* serovar Heidelberg outbreak, Australia, 2018–2019.
