# Supplementary material for: Multistate Outbreak of Salmonella enterica Serovar Heidelberg with Unidentified Source, Australia, 2018–2019
Source: Emerg Infect Dis. 2022 Jan;28(1):238–41. doi: 10.3201/eid2801.211462 (PMC8714228; doi:10.3201/eid2801.211462)
Supplement: Appendix — Additional information about Salmonella enterica serovar Heidelberg outbreak, Australia, 2018–2019. [file 21-1462-Techapp-s1.pdf]

Article DOI: https://doi.org/10.3201/eid2801.211462

Multistate Outbreak of *Salmonella enterica* Serovar Heidelberg with Unidentified Source, Australia, 2018–2019

Appendix

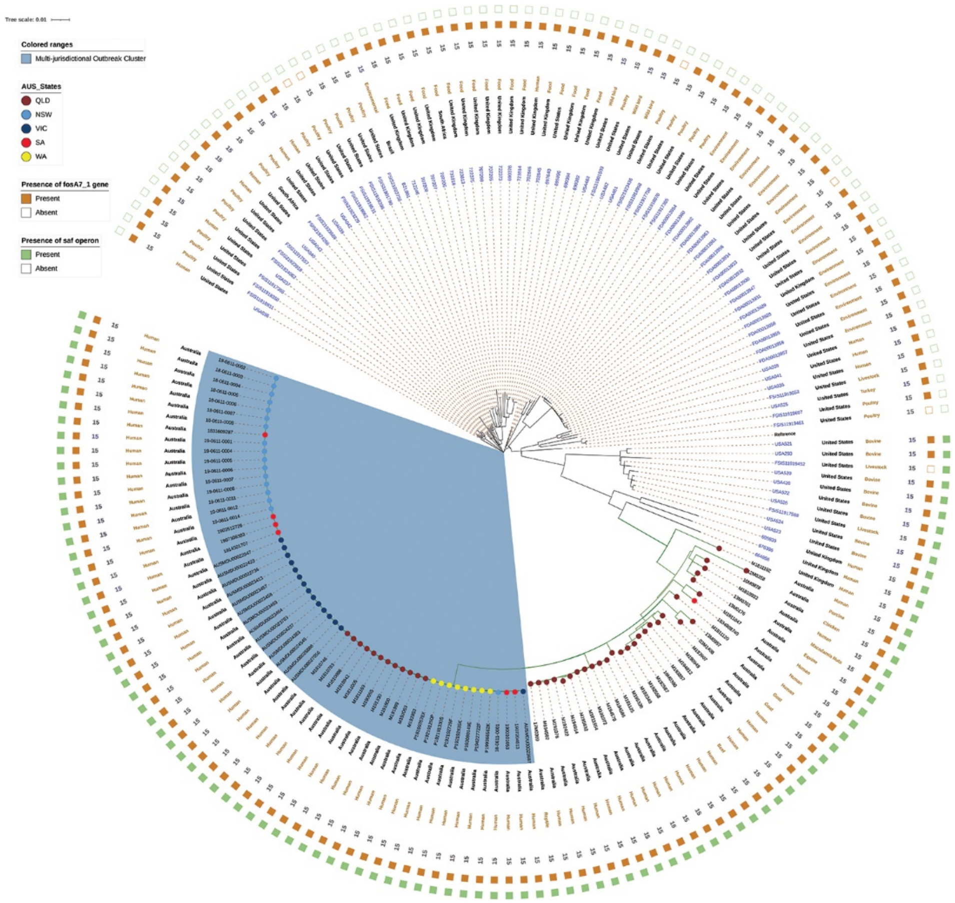

Appendix Figure. Phylogenetic maximum-likelihood tree built using SNP differences between isolates. Green branches and black isolate ID represent the Australia isolates, whereas black branches and blue isolate ID represent the international isolates. Blue shading indicates the isolates belong to multijurisdictional outbreaks. Country of isolation, source, sequence type, presence of *fosA7* gene, and presence of *saf* operon are also included in the tree from inner to outer ring after the isolate ID. Branch length represents distance between groups as indicated by the scale bar. SNP, single nucleotide polymorphism; ST, sequence type.
